# A constitutional isomer selective chemical proteomic strategy for system-wide profiling of protein lysine 5-hydroxylation†

**DOI:** 10.1039/d4sc05397d

**Published:** 2024-10-08

**Authors:** Yi-Cheng Sin, Meeyeon Park, Timothy J. Griffin, Jeongsik Yong, Yue Chen

**Affiliations:** a Department of Biochemistry, Molecular Biology and Biophysics, University of Minnesota at Twin Cities Minneapolis Minnesota USA YueChen@umn.edu jyong@umn.edu; b Bioinformatics and Computational Biology Program, University of Minnesota at Twin Cities Minneapolis Minnesota USA

## Abstract

Lysine 5-hydroxylation (5-Hyl) has been well recognized as an essential protein post-translational modification regulating cellular structural stability, RNA alternative splicing and epigenetic gene expression. System-wide enrichment and quantification of 5-Hyl targets have been challenging due to their chemical inert nature and difficulties in differentiating structural isomers in a complex biological sample. Here, we report the development of an efficient chemical proteomic workflow for affinity enrichment and constitutional isomer specific profiling of endogenous 5-Hyl substrates based on highly selective periodate chemistry. Our study confidently identified over 1600 5-Hyl sites on 630 proteins in human 293T cells, revealing functional significance of the modification in protein structure, transcription and chromatin regulation. Analysis of histone 5-Hyl sites showed that histones H2B and H1 are major targets of the 5-hydroxylysine epigenetic mark. Quantitative proteomic analysis through our chemical enrichment workflow identified specific 5-Hyl substrate proteins mediated by the overexpression of Jumonji-domain containing protein 6 (JMJD6). Our study uncovered two cancer-relevant alternative splice isoforms of JMJD6 that regulate 5-Hyl proteins in distinct cellular pathways, providing unique insights into the functional roles of JMJD6 alternative splicing in transcriptional regulation and cellular development.

## Abstract 2

An efficient chemical proteomic strategy has been developed for affinity enrichment and constitutional isomer specific identification of protein lysine 5-hydroxylation (5-Hyl) based on highly selective periodate chemistry.

## Graphical abstract

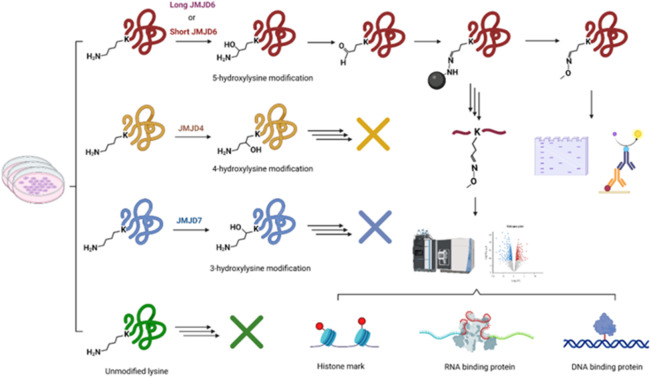

## Introduction

As an ancient and evolutionarily conserved protein post-translational modification (PTM), lysine hydroxylation (Hyl) is broadly involved in protein structural maintenance, RNA processing and epigenetics regulations.^1–5^ Pioneering studies showed that lysine hydroxylation products can be further classified into 5*R*-hydroxylysine, 5*S*-hydroxylysine, 4-hydroxylysine and 3*S*-hydroxylysine based on structural characteristics, mediated by several families of enzymes including PLOD proteins (procollagen-lysine and 2-oxoglutarate 5-dioxygenases), JMJD4 (Jumonji domain containing protein 4), JMJD6 (Jumonji domain containing protein 6) and JMJD7 (Jumonji domain containing protein 7).^2–8^ Among all the constitutional isomers, lysine 5-hydroxylation (5-Hyl) was first discovered and known to be mediated by the PLOD family and JMJD6 protein.^2,3^

JMJD6 is a JmjC family nuclear protein with both arginine demethylation and lysine hydroxylation activities regulating RNA alternative splicing processes, cancer progression and embryo development.^3,4,9–12^ According to the TCGA SpliceSeq database, tumors compared to normal tissues may have different preference in the alternative termination of JMJD6.^13^ For example, tumor samples in glioblastoma (GBM) showed 86.4 PSI (Percent Spliced-In) at exon 7.2 and 13.4 PSI at exon 8. In contrast, normal tissue samples in the same dataset exhibit approximately 47 PSI at exon 7.2 and 53 PSI at exon 8.^13^ The different alternative termination of the JMJD6 gene results in different splicing isoforms but the functional difference of these splicing isoforms is not known.

Due to the lack of affinity enrichment strategies and tools to differentiate constitutional isomers of hydroxylysine, the classic approach to identify 5-Hyl typically involved mass spectrometry (MS)-based identification of the candidate 5-Hyl protein, followed by *in vitro* enzymatic reactions, peptide hydrolysis and HPLC separation of Hyl isomers for comparisons with synthetic standards.^4,6,8^ This strategy provided confident identification of 5-Hyl *in vitro* but cannot directly identify endogenous 5-Hyl in a site-specific manner. Recently, a chemical labeling strategy was developed by applying imidate to label 5-Hyl followed by selective hydrolysis with ammonia.^14^ Though the strategy could label 5-Hyl, it also readily reacts with unmodified lysine and cysteine. Moreover, their chemistry cannot differentiate 5-, 4- or 3-hydroxylysine modification through the ring structure by the imidate chemistry. In addition, the selectivity of the strategy requires the hydrolysis of the labeling on unmodified lysine in ammonia for one hour at 90 °C, a condition that may induce unwanted side reactions in complex protein mixtures. A more recent study applied JMJD6 affinity enrichment followed by a pan-propionylation chemical proteomic approach to identify hydroxylysine targets based on interaction with JMJD6.^9^ However, the exact isomer structure of the modified hydroxylysine was still uncertain.

In this study, we applied periodate oxidation with a highly efficient capturing and releasing strategy for constitutional isomer-selective enrichment and site-specific identification of protein 5-Hyl targets in complex biological samples. Our study reported 1618 human 5-Hyl sites with high confidence and revealed 5-Hyl modifications on RNA binding proteins, histones and transcription factors. Also, we introduced a strategy to enable label-free quantitation of 5-Hyl induced by JMJD6 splicing isoforms and revealed the isoform-specific enrichment of 5-Hyl substrates in diverse biological pathways. Our chemical proteomic strategy and the large coverage of 5-Hyl targets provide unique insights into the mechanisms and regulations of 5-Hyl pathways in cells.

## Results

### Constitutional isomer selective chemical reaction of the 5-hydroxylysine modification

**Fig. 1:**
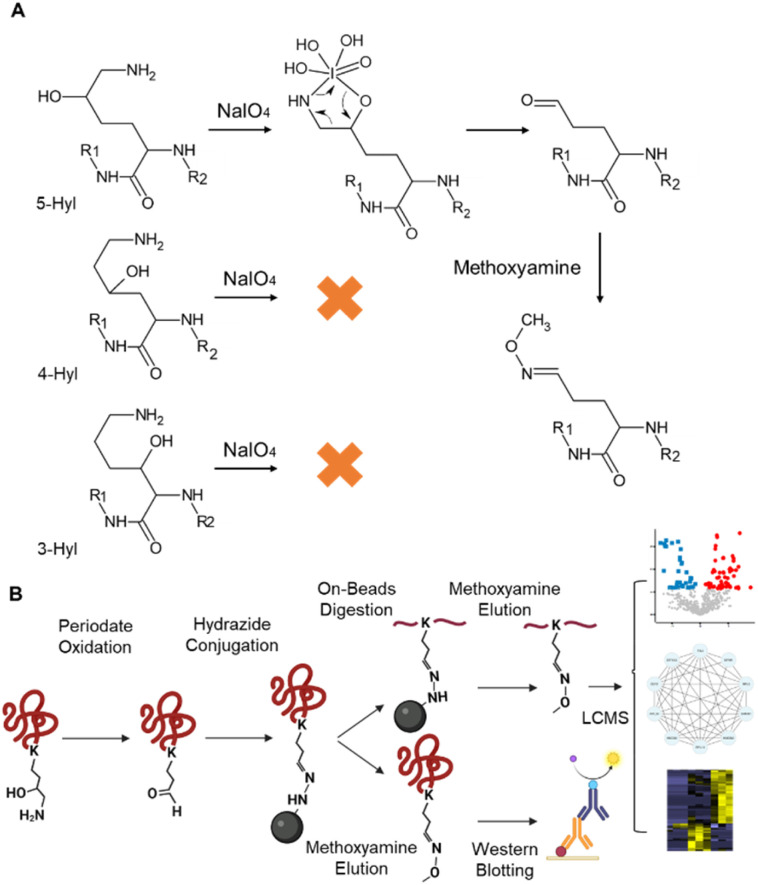
Constitutional isomer selective enrichment and analysis of protein 5-Hyl modification. (A) Chemistry of the constitutional isomer selective reaction of protein lysine 5-hydroxylation. (B) Enrichment and analysis workflow to systematically profile protein 5-Hyl modification.

Periodate-cleavage of vicinal diol groups has been widely applied in glycobiology for global and site-specific analysis of protein glycosylation.^15–18^ Given the chemical similarity between vicinal diol and the lysine side chain with 5-hydroxylation, we reasoned that periodate-based chemistry can selectively oxidize the side chain of 5-Hyl on proteins to aldehyde but not for other constitutional isomers of hydroxylysine, which would enable constitutional isomer selective identification of protein 5-Hyl (Fig. 1A). The oxidized 5-Hyl-containing peptides or proteins with a reactive aldehyde functional group on the side chain can be further conjugated to hydrazide beads for affinity enrichment and released by Schiff-base formation with methoxyamine for detection with mass spectrometry or western blotting (Fig. 1B).

**Fig. 2:**
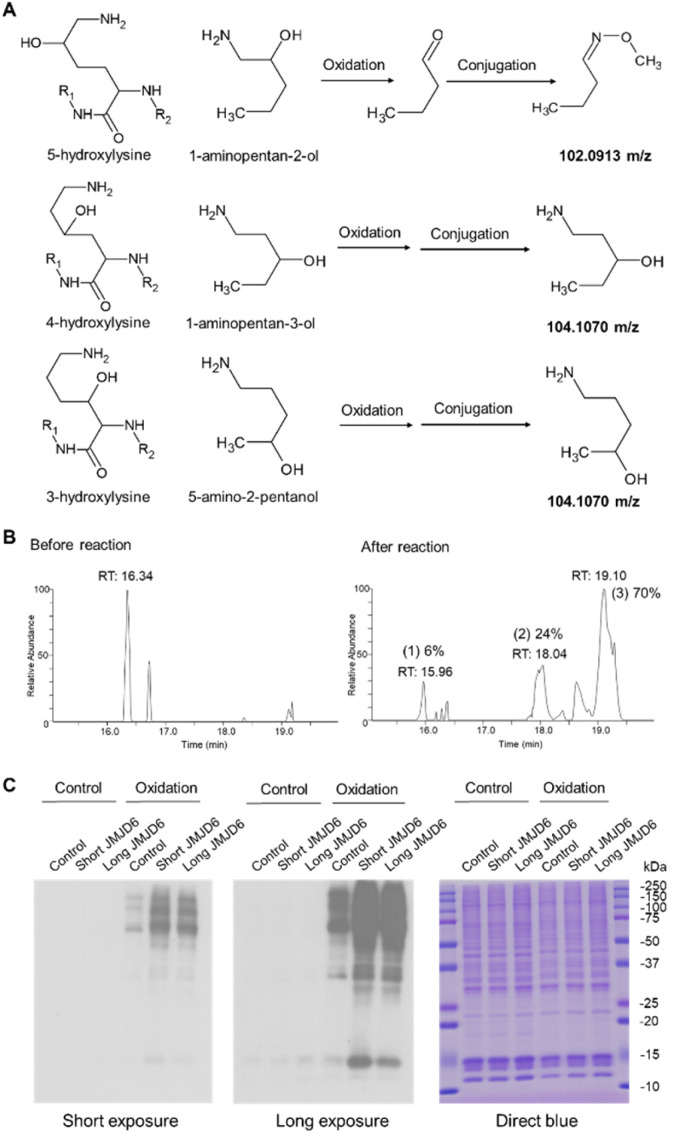
Validation of the constitutional isomer selective chemical reaction to analyze protein 5-Hyl modification. (A) Validation of the constitutional isomer selective chemical reaction of protein 5-hydroxylysine, 4-hydroxylysine and 3-hydroxylysine modification through small molecular chemistry. (B) Validation and evaluation of the reaction efficiency through the tryptic digests of calf collagen. Collagen peptide “GFPGTPGLPGF**K**GIR” (**K** indicates the known 5-Hyl site) was monitored before and after the reactions with periodate oxidation and methoxyamine conjugation. After reactions, extracted ion chromatographic peak areas of peptide forms that were unreacted (782.9080–782.9100 *m*/*z*) (1), periodate-oxidized (767.3880–767.3900 *m*/*z*) (2) and methoxyamine-conjugated (781.9005–781.9024 *m*/*z*) (3) were analyzed and the percentages of peak areas of each peptide form were calculated. (C) Streptavidin-HRP blot detection and validation of protein 5-Hyl modification induced by JMJD6 overexpression. Nuclear lysates were prepared from cells with or without overexpressing JMJD6 long or short isoforms and proteins with or without periodate oxidation were subjected to biotin hydrazide labeling and streptavidin-HRP blot detection.

To validate the specificity of this chemical strategy, we obtained commercially available chemicals, 1-aminopentan-2-ol, 1-aminopentan-3-ol, and 5-amino-2-pentanol that are structurally identical to the side chains of 5-hydroxylysine, 4-hydroxylysine, and 3-hydroxylysine, respectively (Fig. 2A) and performed periodate oxidation followed by methoxyamine labeling. Analysis of these reaction products showed that only 1-aminopentan-2-ol which is structurally similar to the side chain of 5-hydroxylysine was readily oxidized and conjugated with methoxyamine, resulting in a change of the precursor ion *m*/*z* from 104.107 *m*/*z* to 102.091, confirming the high selectivity of the periodate reaction for 5-hydroxylysine (Fig. S1†).

### Efficient labeling and detection of lysine 5-hydroxylation in peptides and proteins

To test if the chemical strategy is applicable in a mixture, we applied this chemical workflow to analyze calf collagen digests, which are known to contain 5-Hyl modification.^19^ To this end, tryptic peptides from collagen were oxidized with periodate and conjugated with methoxyamine, followed by LC-MS/MS analysis for site-specific identification of modified peptides (Fig. S2A†). From the analysis, we identified the COL1A2 K175 site, a known 5-Hyl site on the peptide “GFPGTPGLPGFKGIR” for targeted quantification (Fig. S2B†).^20^ Comparing the percentages of chromatographic peak areas of the unreacted, oxidized and methoxyamine-conjugated peptide forms in LCMS analysis after reactions showed that the efficiency of oxidation was 94% (sum of the peak area percentages of oxidized and conjugated peptide forms) and the efficiency of conjugation was over 70% (Fig. 2B). Based on the potential gas-phase chemical reaction, we further deduced the neutral loss signature for peptides with lysine side chains modified with either aldehyde or methoxyamine conjugation that served as a diagnostic indicator of the modification (Fig. S3†). This neutral loss feature was further validated by comparing the spectra of the D_3_- and H_3_-methoxyamine conjugated peptides with that of *in vivo* 5-Hyl (Fig. S4†).

Using biotin-hydrazide instead of methoxyamine for conjugation, we performed streptavidin blot to validate the selectivity and efficiency of our labeling strategy. We prepared nuclear lysates from cells transfected with either control flag plasmids or N-terminal flag-tagged long and short splicing isoforms of JMJD6 plasmids corresponding to the JMJD6 isoforms with termination at Exon 8 and 7.2, respectively (Fig. S5A†). Indeed, our analysis showed that expression of either JMJD6 isoform globally and significantly increased lysine 5-hydroxylation, confirming the efficiency of our chemical strategy (Fig. 2C and S5B†). The strong blot intensity at the molecular weight of 10–15 kDa suggested that JMJD6 expression may induce 5-Hyl modification on histones.

### Chemical proteomic workflow enabled global profiling of 5-Hyl sites in human cells

**Fig. 3:**
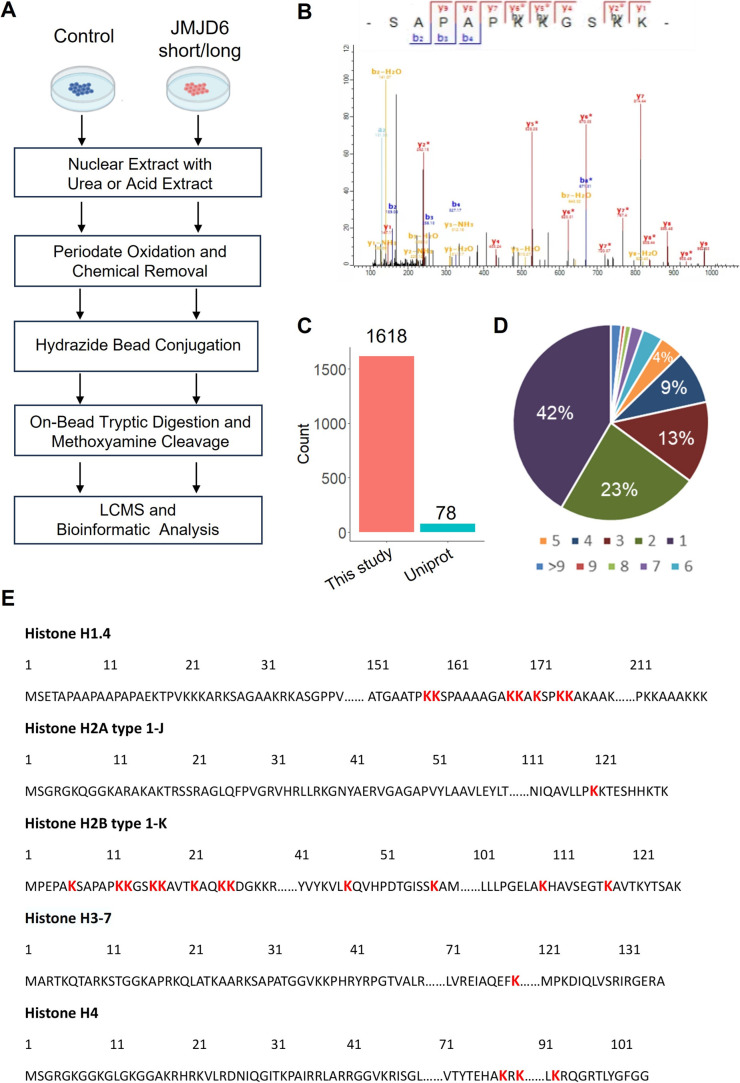
Chemical proteomic profiling of lysine 5-hydroxylation modification on nuclear proteins in human cells. (A) An experiment workflow for the site-specific detection of protein 5-Hyl modification. (B) An example spectrum of the identified 5-Hyl-containing histone H2B peptide. ‘Hy’ indicates methoxyamine-conjugated oxidized 5-Hyl modification. ‘*’ indicates the neutral loss of methoxyamine conjugated oxidized 5-Hyl modification. (C) Comparison of identified 5-Hyl sites in this study to 5-Hyl sites annotated in the Uniprot database. (D) A pie chart for the percentage of 5-Hyl site counts per protein. (E) A map of 5-Hyl identifications on core histones and histone H1 in this study. Identified 5-Hyl sites were highlighted in red font and only a single variant for each type of histone protein is shown as an example.

To enable global proteomic profiling of 5-Hyl modification, we developed a streamlined chemical proteomics strategy based on the specific reaction of 5-Hyl oxidation and a highly efficient capturing and releasing strategy (Fig. 3A). We coupled the strategy with the overexpression of JMJD6 isoforms to systematically profile 5-Hyl modification sites in mammalian cells. Briefly, 293T cells expressing either control plasmids or plasmids encoding either the long or short form of JMJD6 were lysed for nuclear extraction as JMJD6 was known to be a nuclear protein. To eliminate the interference of the small molecules and oligonucleotides, we optimized our workflow by performing benzonase digestion of nucleotides and filter-based metabolite removal. Proteins were then oxidized with periodate and about 1 mg of proteins was subjected to incubation with hydrazide-beads. Following incubation, the beads were extensively washed. Conjugated proteins were digested on beads with trypsin followed by a second wash to remove non-conjugated peptides. Finally, conjugated peptides were eluted off the beads with competitive methoxyamine elution and desalted for LCMS analysis (Fig. 3A). For specific characterization of histone 5-Hyl sites, we performed acid extraction of nuclear pellets from 293T cells expressing short-form JMJD6. Extracted proteins were subjected to a similar procedure for the enrichment and identification of 5-Hyl targets as the analysis of nuclear lysates (Fig. 3A).

Our systematic profiling confidently identified a total of 1618 5-Hyl sites on 630 proteins, representing the largest coverage of the 5-Hyl proteome to date with constitutional isomer specificity (Fig. 3B, C, S6, Tables S1 and S2†). Nearly 60% of the proteins were identified with more than one site and nearly 10% were identified with ≥6 sites, suggesting the preference of 5-Hyl to target multiple lysines on the same substrate (Fig. 3D).

Histones were found to be extensively modified with 5-Hyl in our analysis with 40 site identifications, which agreed with the observations in the streptavidin blot (Fig. 3E, Tables S1 and S2†). Interestingly, most of the histone 5-Hyl sites were identified on variants of histones H2B and H1, which is in distinct contrast to other well-known histone epigenetic marks such as acetylation and methylation that are abundant on histones H3 and H4 (Fig. 3E and S7†). Even including lysine acetylation as a variable modification in data analysis did not boost the identification of 5-Hyl sites on histones H3 and H4. Notably, the 5-Hyl sites on histones H3 and H4 were only identified in the histone-enriched acid extraction samples with low spectral counts. These data suggested that histones H2B and H1 are major targets of histone lysine 5-hydroxylation. In this study, we also identified known JMJD6 targets U2AF2 and BRD4 (ref. 3 and 9) with K70 sites on U2AF2 and nine sites on BRD4 (Tables S1 and S2†).

To evaluate if our workflow could also identify O-GlcNAc-containing proteins as previously suggested,^21^ we analyzed our data by specifying methoxyamine-conjugated O-GlcNAc modification as variable modifications for Ser and Thr. Indeed, we identified over 1200 O-GlcNAc modification sites from the nuclear lysate dataset, suggesting that our chemical proteomics workflow can be efficiently applied to simultaneously study multiple PTMs in a streamlined manner (Table S3†).

### Bioinformatics analysis revealed structural characteristics and functional enrichment of protein 5-Hyl targets in human cells

**Fig. 4:**
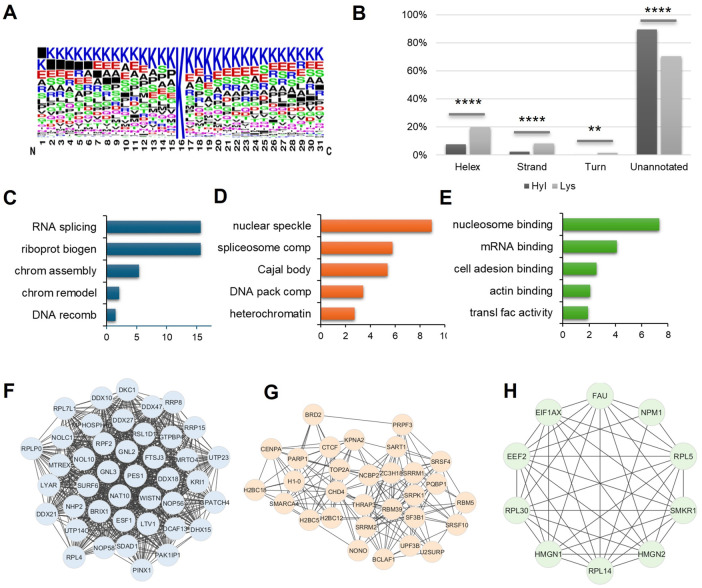
Bioinformatic characterization of 5-Hyl proteins from the system-wide profiling analysis. (A) Flanking sequence analysis of protein 5-Hyl modification sites identified in this study. (B) Secondary structure distribution analysis of protein 5-Hyl modification sites (dark grey bars) compared to all lysines among identified peptides in this study (light grey bars). **** indicates *p* < 0.0001 in the hypergeometric test. (C–E) Gene ontology annotation enrichment analysis of 5-Hyl proteins in this study on (C) biological processes, (D) cellular compartment and (E) molecular function. (F–H) Network representation of highly connected clusters of 5-Hyl proteins with (F) a subnetwork related to ribonucleoprotein biogenesis, (G) a subnetwork related to transcription and DNA/RNA binding and (H) a subnetwork related to the ribonucleoprotein complex.

To determine the enrichment of structure features and biological pathways, we performed bioinformatic analysis of 5-Hyl protein targets identified in the total nuclear lysate analysis. Flanking sequence motif analysis showed the strong preference of 5-Hyl on lysine-rich regions with some preferences for glutamic acid, serine, alanine and proline in the close vicinity of the modified lysine (Fig. 4A). Aligning identified 5-Hyl sites with experimentally validated secondary structures in the Uniprot human database showed that only 10% of 5-Hyl sites were annotated in the secondary structures with 8% localized to alpha-helix and 2% localized to the beta strand, while 30% of all lysines in tryptic peptides were annotated in secondary structures with 20% localized to alpha-helix, 8% localized to the beta strand and 2% localized to turns (Fig. 4B). These data indicated a significant depletion of 5-Hyl on protein regions with secondary structures.

Pathway and gene ontology enrichment analysis showed that 5-Hyl modified proteins were significantly enriched in pathways and processes related to RNA processing and chromatin remodeling with cellular localization enrichment in nuclear speckle, spliceosome and heterochromatin (Fig. 4C–E and Table S3A–C†). Interaction network analysis identified multiple highly connected interaction subnetworks including the ribonucleoprotein biogenesis network, the transcription network and ribonucleoprotein complexes (Fig. 4F–H). Hence, our analysis of 5-Hyl modified proteins suggested the critical role of modification in RNA processing and chromatin-related activities in human cells.

### Differential analysis of JMJD6 long-form and short-form regulated 5-Hyl sites

**Fig. 5:**
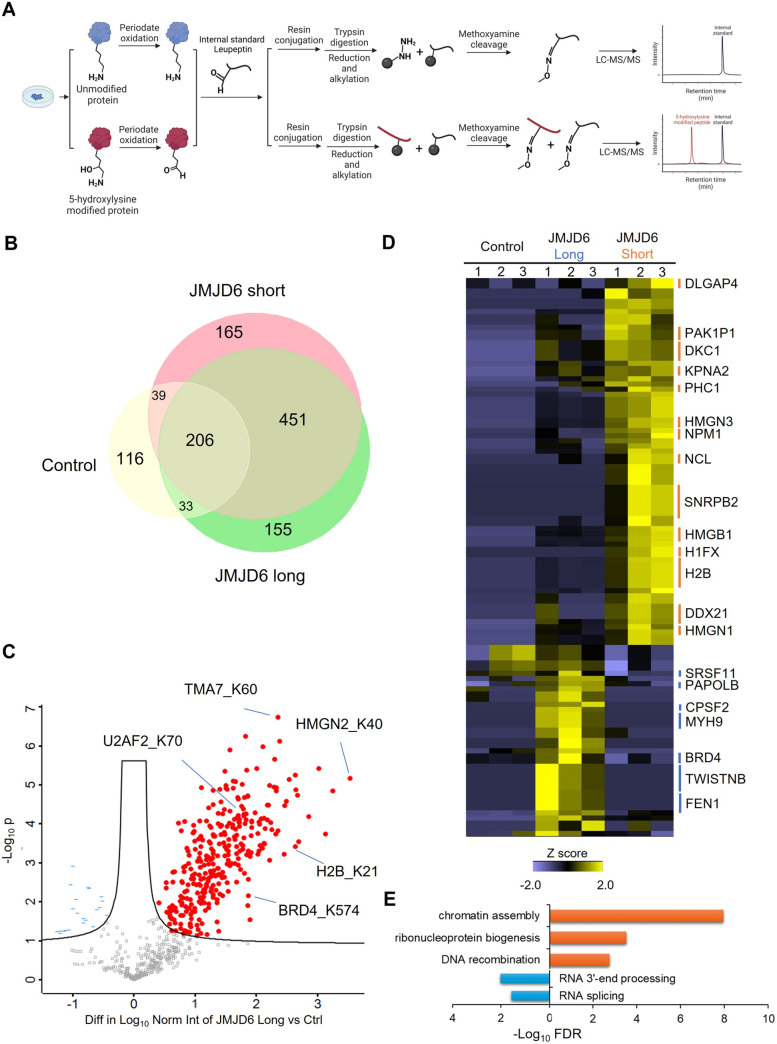
Quantitative analysis of 5-Hyl modification sites in nuclear lysate analysis mediated by JMJD6 expression. (A) A scheme for the label-free quantification of site-specific 5-Hyl with a spike-in internal standard. (B) A Venn diagram showing the overlap of protein 5-Hyl identification with or without the expression of JMJD6 splicing isoforms. (C) A volcano plot showing the dynamic changes in 5-Hyl abundances upon the expression of N-terminally flag-tagged JMJD6 long isoform (two-sample Student's *T*-test with FDR <0.05). (D) A heatmap and clustering analysis of significantly changed 5-Hyl sites induced by the overexpression of either the long or short form of JMJD6. Normalized label-free quantification of significantly changed 5-Hyl targets (*p* < 0.05, one-tail Student's *T*-test analysis assuming unequal variance) were *Z*-score converted and clustered with hierarchical clustering. (E) Gene ontology biological process enrichment analysis of 5-Hyl proteins differentially induced by the expression of specific JMJD6 isoforms (orange bars: short-form and blue bars: long-form).

For quantitative analysis of JMJD6-regulated 5-Hyl sites, we applied a label-free quantification strategy with an aldehyde-containing peptide, leupeptin, as a spike-in internal standard after periodate oxidation (Fig. 5A). By normalizing the identified 5-Hyl peptide peak intensity to the methoxyamine conjugated leupeptin, this strategy eliminated quantification variations in the enrichment process such as variations in the efficiencies of Schiff-base formation and cleavage as well as inconsistent loss during sample processing.

Our systematic profiling showed that overexpression of either the long or short form of JMJD6 led to an apparent increase in the total number of 5-Hyl site identifications in nuclear lysate analysis with 845 sites identified from long form expression and 861 sites identified from short form expression, compared to 394 sites identified from the control plasmid expression (Fig. 5B). The large overlap between these analyses demonstrated excellent reproducibility in identifying common 5-Hyl modification sites. We further performed the label-free quantitative analysis with internal standard normalization. The volcano plot analysis identified 283 sites and 303 sites that were significantly upregulated with the overexpression of the JMJD6 short form and long form, respectively (two-sample Student's *T*-test with an FDR threshold of 0.05) (Fig. 5C and S8†). Given the well-recognized role of JMJD6 in transcription and RNA processing, it is important to note that the upregulation of specific 5-Hyl site abundance upon the expression of JMJD6 isoforms could not only be due to direct JMJD6 hydroxylase activities, but also result from JMJD6-mediated transcriptional activities or potentially other 5-Hyl enzymes regulated by JMJD6.

Quantitative comparison of 5-Hyl abundance from the expression of long or short JMJD6 isoform identified a group of 108 sites, among which 71 sites showed a stronger increase in abundance with the expression of the short-form JMJD6, while 37 sites showed a stronger increase in abundance with the expression of the long-form JMJD6 (Fig. 5D and Table S5†). Pathway and functional annotation enrichment analysis showed that 5-Hyl proteins with a higher abundance by the short-form JMJD6 expression were significantly enriched in chromatin assembly (adj. FDR <1.3 × 10^−8^) and ribonucleoprotein complex biogenesis (adj. FDR <3.2 × 10^−4^) processes (Fig. 5E). On the other hand, 5-Hyl proteins with a higher abundance upon the long-form JMJD6 expression were significantly enriched in RNA 3′-end processing (adj. FDR <0.3 × 10^−3^) and RNA splicing (adj. FDR <2.7 × 10^−2^) (Fig. 5E). Interestingly, motif enrichment analysis showed that 5-Hyl sites identified by the overexpression of the short-form JMJD6 harbored a unique “PK” motif which likely correlates with structural characteristics and substrate interaction of the short-form JMJD6 (Fig. S9†). Taken together, these data clearly demonstrated that splicing isoforms of JMJD6 have differential preferences for regulated 5-Hyl sites and aberrant expression of JMJD6 splicing isoform under certain pathophysiological conditions may affect the 5-Hyl substrates and activities in specific cellular pathways.

### Chemical pulldown assay and western blotting analysis of 5-Hyl modification proteins regulated by JMJD6

**Fig. 6:**
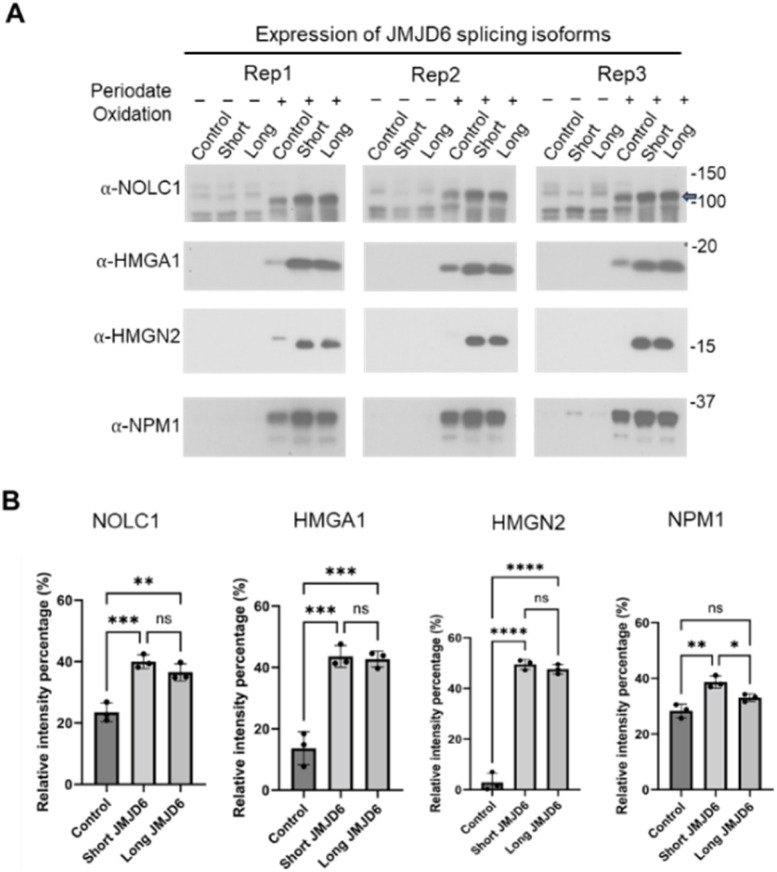
Chemical pull-down assay to validate JMJD6-regulated 5-Hyl proteins. 293T cells were transfected with either a control plasmid or a flag-tagged JMJD6 short or long form plasmid. Proteins were pulled down with hydrazide beads following periodate oxidation and eluted using the methoxyamine buffer. (A) Western blotting analysis of selected 5-Hyl proteins, NOLC1, HMGA1, HMGN2 and NPM1. (B) Quantification of the western blotting analysis from the chemical pulldown assay with one-way ANOVA. *p < 0.05, **p < 0.01, ***p < 0.001, and ****p < 0.0001.

To further confirm these findings, we performed a chemical pulldown assay with periodate oxidation of 5-Hyl proteins, hydrazide-bead conjugation and methoxyamine elution followed by western blotting detection (Fig. S10†). From the list of our identifications, we selected NOLC1, HMGN2, HMGA1 and NPM1 as examples where each of these proteins was identified with high quality MS/MS spectra and confident site localizations (Fig. S11A–D†). Our western blots showed that these proteins showed minimal signals from the pulldown assay without oxidation regardless of the overexpression conditions. Upon oxidation and pulldown, each of these proteins was clearly captured, enriched and detected. Expression of either the long or short form of JMJD6 led to a significant increase among each pulled-down target, consistent with our label-free quantitative analysis of NOLC1, HMGN2 and HMGA1 (Fig. 6). Interestingly, NPM1 showed a consistently stronger intensity in the chemical pulldown assay upon the expression of the short-form JMJD6 compared to the expression of the long-form JMJD6 across multiple biological replicates, confirming that the expression of short and long forms of JMJD6 have differential regulations towards the overall NPM1 5-Hyl abundance *in vivo* (Fig. 6).

## Conclusions and discussion

In this study, we developed an efficient chemical proteomics workflow for global enrichment and constitutional isomer specific profiling of 5-Hyl protein modification substrates based on highly selective periodate chemistry. Confident identification of 1618 5-Hyl sites on 630 proteins represented the largest coverage of the modification in human cells with constitutional isomer specificity and suggested that lysine 5-hydroxylation is a widespread PTM broadly involved in chromatin and RNA processing activities in addition to the canonical view of its important functions related to collagen crosslinking and glycosylation. Interestingly, we observed significant depletion of 5-Hyl on the secondary structure indicating a potentially important role of the modification in protein folding and structural stability. Application of label-free quantitative analysis demonstrated the *in vivo* activity of JMJD6 and allowed us to confidently identify over 300 potential JMJD6 regulatory sites. Importantly, with our strategy, we discovered that long and short alternative splicing forms of JMJD6 exhibit differential activities towards specific proteins or signaling pathways in cells. A technical limit of the current study was that we could not determine whether the 5-Hyl sites with increased abundance with JMJD6 overexpression were JMJD6 direct hydroxylation targets. Further technology development by integrating this chemical proteomics workflow with isotopic-labeling strategies such as SILAC and TMT multiplexing^22,23^ and quantitative JMJD6 interactome analysis^9^ will reveal novel insights into direct JMJD6 targets in mammalian cells.

Our study revealed many novel targets as 5-Hyl substrates. Histones are highly abundant nuclear proteins and decorated with over 10 types of PTMs for epigenetic regulation of gene expression and the chromatin structure.^24^ We identified histone lysine 5-hydroxylation with both streptavidin blot and MS analysis. Surprisingly, we found that histones H2B and H1 are major targets of 5-Hyl modification, suggesting the potentially distinct role of 5-Hyl as an epigenetic mark compared to other well-studied histone modifications on histones H3 and H4 such as acetylation and methylation. From our analysis, we also identified eukaryotic peptide chain release factor subunit 1 (ETF1) K63 as a 5-Hyl target (Fig. S11E†). Interestingly, a previous study showed with *in vitro* enzymatic reactions that ETF1 K63 is targeted by JMJD4 for 4-hydroxylysine modification to regulate translational termination efficiency.^6^ Our analysis demonstrated that ETF1 can be targeted for 5-Hyl *in vivo* as a potentially alternative mechanism to regulate translational termination.

JMJD6 is increasingly recognized for its multifaceted role in the pathogenesis of various diseases, particularly cancer, due to its enzymatic functions as a lysyl hydroxylase and arginine demethylase.^25–29^ These activities are crucial for modulating RNA splicing, transcription, and signal transduction pathways. Our study, highlighting differential activities among JMJD6 isoforms, introduces a novel aspect to our understanding of its pathogenic roles. This discovery not only enhances our grasp of JMJD6-driven mechanisms but also sharpens our focus on identifying precise molecular targets, potentially unveiling novel therapeutic strategies and elucidating previously unrecognized pathogenic pathways. Furthermore, our chemical MS-based proteomic workflow should be applicable to characterization of other enzyme mediators of this important protein PTM.

## Experimental section

### Chemicals and reagents

Anti-B23/NPM1 polyclonal antibody (10306-1-AP), anti-HMGN2 antibody (10953-1-AP), anti-HMGA1 polyclonal antibody (29895-1-AP), anti-JMJD6 antibody (16476-1-AP) and anti-NOLC1 polyclonal antibody (11815-1-AP) were purchased from Proteintech (Billerica, MA). Anti-rabbit IgG (HRP-linked) antibody and anti-DYKDDDDK Tag antibody (D6W5B) were from Cell Signaling (Danvers, MA). Ultralink hydrazide resins (53149) and sodium *meta*-periodate (PI20504) were from ThermoFisher Scientific (Waltham, MA). Methoxyamine hydrochloride (SIAL-226904), (+)-biotin hydrazide (B7639) and benzonase (70746-4) were from Sigma-Aldrich (St. Louis, MO). Methoxyl-d3-amine HCl (D-1272) was from CDN Isotopes (Pointe-Claire, Quebec, Canada). Leupeptin hemisulfate (L22035-0.005) was from RPI (Mount Prospect, IL). HRP-labeled streptavidin (405210) was from BioLegend (San Diego, CA). The FLAG-HA-pcDNA3.1 plasmid was a kind gift from Adam Antebi (Addgene # 52535). 5-Amino-2-pentanol (ST0059AI) was from 1st Scientific (San Diego, CA). 1-Aminopentan-3-ol (CSSB00000748770) and 1-aminopentan-2-ol (CSSB00000755064) were from ChemSpace (Monmouth Junction, NJ).

### Plasmid preparation for JMJD6 splicing variants

Human JMJD6 isoforms were cloned into pCMV10 (addgene plasmid #47334) for N-terminal flag-epitope tagging by PCR using the following primer sets. For the JMJD6 forward primer, 5′-GATAAAGCTTAACCACAAGAGCAAGAAGCGCATCCG-3′ was used. For the short form JMJD6 isoform construction (ENST00000397625.9), a reverse primer 5′-ATTATCTAGATCACCTGGAGGAGCTGCGCTCTTTGCTGAC-3′ was used. For the long form JMJD6 isoform construction (ENST00000445478.6), the 5′-ATTATCTAGATCAGGGGTGAGCCCGGCCTCCACAAGTGTCC-3′ reverse primer was used. Total RNAs from HEK293 cells were isolated and oligo d(T) primed cDNA synthesis was done. Then, PCR amplification using the above primers was conducted for cloning into pCMV10.

### Cell culture and transient transfection

293T cells (ATCC #CRL-3216) were cultured at 37 °C with 5% CO_2_ and maintained in Dulbecco's Modified Eagle Medium (DMEM) (ThermoFisher Scientific, Waltham, MA), supplemented with 10% fetal bovine serum (Sigma, St. Louis, MO), 100 IU penicillin, and 100 μg ml^−1^ streptomycin (Genesee Scientific, Morrisville, NC). Cells were seeded in a 15 cm plate overnight and were then transfected with 1 ml of premixed medium containing 45 μg polyethyleneimine (PEI) (Sigma, St. Louis, MO) and 20 μg plasmid for 48 hours.

### Reactivity analysis with chemical compounds

Aminopentanol at a final concentration of 25 mM was mixed with 75 mM sodium periodate in 0.25 M sodium acetate solution pH 4.5 and incubated in the dark for 5 min. Then, an equal volume of 200 mM methoxyamine was added to the sample and incubated at room temp. for 5 minutes. Finally, 20% volume of acetonitrile was added to the sample and the sample was infused to an Exactive Orbitrap mass spectrometer (ThermoFisher Scientific, Waltham, MA) with a positive mode full scan (90–110 *m*/*z*) and 25 000 resolution (FWHM) at *m*/*z* 400.

### Hydroxylysine reactivity analysis with collagen

Calf collagen (Sigma, St. Louis, MO) was dissolved in 9 M urea with 100 mM TEAB and centrifuged to remove the pellet. The dissolved collagen solution was added to TCEP and IAA to a final concentration of 10 mM for each with rotation at room temp in the dark for 30 min for reduction and alkylation. Then, samples were added to 20 mM beta mercaptoethanol to quench the reaction. The solution was diluted with water to 1.5 M Urea and subjected to trypsin digestion. The digested protein solution was further desalted using an Oasis C18 cartridge (Waters, Milford, MA) and reconstituted into 200 mM sodium acetate pH 4.5 solution. The samples were centrifuged to remove the pellet and incubated with 50 mM sodium periodate in 200 mM sodium acetate pH 4.5 in the dark at room temp with rotation for 30 min. The oxidized peptides were desalted using Pierce™ C18 desalting tips (ThermoFisher Scientific, Waltham, MA). Finally, the desalted oxidized peptides were incubated with 200 mM methoxyamine 200 mM sodium acetate pH 4.5 at room temp for 30 min with rotation and desalted using home packed StageTips for LCMS analysis.

### Nuclear lysate preparation for chemical labelling

Cells were briefly rinsed and scraped down with PBS and were lysed in the hypotonic buffer (Active motif, Carlsbad, CA) with 0.5% NP40 and 1 mM PMSF (ThermoFisher Scientific, Waltham, MA) and incubated on ice with manual rotation from time to time for 10 minutes followed by centrifugation at 16 000×*g* to obtain the nuclear pellets. The nuclear pellets were then lysed with the urea lysis buffer (9 M urea with 1 mM PMSF) and subjected to sonication (45%, 10 cycles, one second on and one second off for each cycle), and centrifugation at 21 000×*g* to obtain soluble nuclear extracts in the supernatant. To remove the oligonucleotide in the sample, the lysate was diluted with water to 3 M urea and treated with 250–290 units of benzonase and 1 mM MgCl_2_ at the final concentration for incubation with rotation at 37 °C for 30 min. Then, the reaction was quenched with 0.5% SDS (w/w) and 50 mM sodium acetate pH 4.5 and the sample was subjected to buffer exchange with water through an Amicon® Ultra-15 centrifugal filter unit (3 kDa) (Sigma, St. Louis, MO). After the buffer exchange, the sample was subjected to oxidation in the periodate oxidation buffer (100 mM sodium periodate, 0.1% SDS (w/w) and 50 mM sodium acetate at pH 4.5) at room temperature with rotation in the dark for an hour. Then, the protein solution was subjected to a second buffer exchange. The protein concentration was measured with the Bradford assay (ThermoFisher Scientific, Waltham, MA).

### Acid extraction for chemical labelling

Cells expressing short form JMJD6-flag were pelleted and lysed in a lysis buffer containing 1× PBS with 0.5% Triton X-100 and 1 mM PMSF (ThermoFisher Scientific, Waltham, MA) for 30 min at 4 °C and centrifuged to obtain the pellet. Then, the pellet was subjected to acid extraction in 0.4 N H_2_SO_4_ overnight. The extracted proteins were precipitated using 20% trichloroacetic acid (TCA) and washed with cold acetone once. The precipitated proteins were dissolved in 0.1% SDS with 100 mM ammonium bicarbonate and subjected to oxidation with 100 mM sodium periodate at room temperature with rotation in the dark for an hour. The oxidized protein was again precipitated using 20% TCA and washed with cold acetone once. Finally, the oxidized protein precipitates were dissolved in 0.1% SDS with 100 mM ammonium bicarbonate and 50 mM sodium acetate (pH 4.5) and incubated with the hydrazide resins for enrichment, followed by on-bead tryptic digestion and elution for LCMS analysis similar to the processing of the nuclear lysate samples.

### Streptavidin blot detection of 5-Hyl proteins

Nuclear protein lysates with or without periodate oxidation were added to biotin hydrazide to a final concentration of 10 mM. Then, samples were further reconstituted in LAEMMLI SDS sample buffer with pH neutralization and proteins were labeled at room temperature with rotation for 30 min. After the incubation, the samples were boiled at 95 °C and subjected to SDS-PAGE analysis for western blot and Imperial™ Protein Stain (ThermoFisher Scientific, Waltham, MA).

### Western blot detection of 5-Hyl proteins with the protein chemical pulldown assay

Nuclear protein lysates with or without periodate oxidation were incubated with the hydrazide resins in acetate buffer (0.1% SDS and 50 mM sodium acetate at pH 4.5) at room temperature overnight with rotation. After the overnight incubation, the supernatant was removed and the beads were subjected to four times washing with 9 M urea, one time washing with 9 M urea in 0.4 M ammonium bicarbonate, one time washing with PBS and one time washing with water. After the washing, the protein conjugated beads were treated with a final concentration of 100 mM methoxyamine in the LAEMMLI SDS sample buffer and incubated at room temperature for 30 min. After the incubation, the sample's pH was neutralized and the sample was then boiled at 95 °C for 5 minutes for SDS-PAGE analysis and western blotting.

### Analysis of 5-Hyl peptides with the protein chemical pulldown assay

One milligram of nuclear proteins from lysates with periodate oxidation was added to 0.5 ng of leupeptin hemisulfate as an internal standard. Proteins were incubated with the hydrazide resins in acetate buffer (0.1% SDS and 50 mM sodium acetate at pH 4.5) with rotation at room temperature overnight. After the supernatant removal, the beads were washed with 9 M urea four times, 9 M urea with 0.4 M ammonium bicarbonate once and PBS once. Then, beads were re-suspended in 100 mM ammonium bicarbonate buffer and the proteins were digested using trypsin with rotation at 37 °C overnight. Following digestion, the beads were washed with PBS four times. Then, we performed the reduction and alkylation on beads with 10 mM TCEP and 10 mM iodoacetamide at the final concentrations in 100 mM ammonium bicarbonate for 30 min with rotation at room temperature in the dark, followed by four times washing with PBS. Finally, the peptides conjugated on beads were eluted using the methoxyamine elution buffer (0.2 M methoxyamine, 0.2 M sodium acetate pH 4.5 and 0.1 M aniline) three times. The eluted fractions were pooled and peptides were desalted for LCMS analysis. Biological triplicate analysis was performed for each treatment condition in quantification.

### NanoHPLC – Orbitrap MS analysis

Peptides were analyzed with a Dionex Ultimate 3000 RSLCnano HPLC system coupled to an Orbitrap Fusion™ Lumos™ Tribrid™ mass spectrometer (ThermoFisher Scientific, Waltham, MA). Digested peptides dissolved in HPLC buffer A (0.1% formic acid in water (v/v)) were separated on an in-house packed capillary HPLC column (length 20 cm and 75 μm inner diameter) from CoAnn Technologies with Luna C18 beads (5 μm particle size, 100 Å pores) from Phenomenex. The peptides were separated with a 30-min (for collage peptide analysis), 60-min (for 5-Hyl site analysis in histone acid extract analysis) or 90-min gradient (for 5-Hyl site analysis in nuclear lysate) from 1% to 95% HPLC buffer B (0.1% formic acid in acetonitrile (v/v)) in HPLC buffer A. Full scan of precursor ions (MS1) was performed in a positive mode and with a resolution of 120 K (at 200 *m*/*z*) in a mass range of 375–1600 *m*/*z*. The linear ion trap with an isolation window of 1.6 *m*/*z* using a quadrupole and a 35% of normalized high-energy collision dissociation (HCD) energy was set for the tandem mass spectral (MS/MS) analysis. Dynamic exclusion for 45 seconds with a mass tolerance of ±10 ppm was enabled for the MS/MS acquisition.

### MaxQuant data analysis for the identification of peptides and proteins

LCMS data was analyzed with the MaxQuant search engine (ver 2.2.0.0) against the Uniprot human reference protein database (UP000005640_9606) concatenated with reversed decoy sequences and common contaminant sequences.^30^ Carbamidomethylation on cysteine was specified as a fixed modification, trypsin was specified as the protease and match between runs was allowed. By default, precursor mass tolerance was set at 4.5 ppm and MS/MS mass tolerance was set at 0.5 Da. A stringent 1% false discovery rate was specified for the identification of proteins, peptides and modification sites with an additional requirement of a minimum score of 40 for the modification site identification. To analyze 5-Hyl modification, the maximum missing cleavage was specified as 6 and the maximum number of modifications allowed per peptides was specified as 6. Variable modifications included oxidized 5-Hyl with methoxyamine conjugation modification (defined as the composition of OH(−2) and a neutral loss of CH(5)NO, and not at the C terminal), protein N-terminal acetylation and methionine oxidation. For the identification of histone 5-Hyl sites that may be co-modified by acetylation, lysine acetylation was included as an additional variable modification. To analyze O-GlcNAc modification, the maximum missing cleavage was specified as 1 and the maximum number of modifications allowed per peptides was specified as 2. Variable modifications included oxidized O-GlcNAc with methoxyamine conjugation modification (defined as the composition of C(10)H(17)N(3)O(10) with a neutral loss of C(10)H(17)N(3)O(10)), protein N-terminal acetylation and methionine oxidation.

### Perseus analysis for label-free quantification

All peptide and protein identifications from the reversed decoy sequences or contaminant sequences or identifications with an Uniprot ID not matching the gene name were removed for label-free quantitative analysis. The total intensity of each modification site was normalized by the chromatographic peak area of methoxyamine-conjugated leupeptin (the spike-in internal standard) of each sample. Then, the data were analyzed with the Perseus bioinformatics platform (version 1.6.15.0)^31^ and statistical analysis performed with two sample Student's *T*-tests with an FDR threshold of 0.05.

### Bioinformatics analysis and software

A flanking sequence motif was generated with Weblogo.^32^ Pathway and gene ontology enrichment analysis were performed with Webgestalt^33^ and statistical significance was specified as an FDR of 0.05 with Benjamini–Hochberg adjustment. The protein–protein interaction network was extracted based on the String database^34^ and visualized with Cytoscape.^35^ Protein clusters with significant connectivity were identified with MCODE.^36^ Heat map and hierarchical clustering analysis was performed with Genesis.^37^ Flanking sequence motif analysis was performed with R package RmotifX.^27^ Schematic figures for workflows were created with https://Biorender.com. The molecular structure was drawn with Chemsketch (2022.2.2, Advanced Chemistry Development, Inc. (ACD/Labs), Toronto, ON, Canada). Bar graph representation with statistical analysis was prepared with GraphPad Prism (GraphPad Software, Boston, Massachusetts, USA). Quantitative image analysis of western blotting was performed with Image J.^38^ Other data analysis and figure presentation was performed with in-house developed R using the libraries (readxl, ggplot2, dplyr, openxlsx, and eulerr) and Python scripts.

## Data availability

The data supporting this article have been included as part of the ESI.† The mass spectrometry proteomics data have been deposited to the ProteomeXchange Consortium *via* the PRIDE^39^ partner repository with the dataset identifier PXD054608.

## Author contributions

YS performed all biochemical, chemical and proteomics studies, MP performed cloning and plasmid preparation, TG, JY and YC supervised the study, and YS and YC wrote the manuscript with inputs from all authors.

## Conflicts of interest

There are no conflicts to declare.

## Supplementary Material

SC-OLF-D4SC05397D-s001

SC-OLF-D4SC05397D-s002

SC-OLF-D4SC05397D-s003

SC-OLF-D4SC05397D-s004

SC-OLF-D4SC05397D-s005

SC-OLF-D4SC05397D-s006
