# Supplementary material for: A constitutional isomer selective chemical proteomic strategy for system-wide profiling of protein lysine 5-hydroxylation
Source: Chem Sci. 2024 Oct 8;15(44):18395–404. doi: 10.1039/d4sc05397d (PMC11474802; doi:10.1039/d4sc05397d)
Supplement: SC-015-D4SC05397D-s001 [file SC-015-D4SC05397D-s001.pdf]

Supplementary Information (SI) for Chemical Science.
This journal is © The Royal Society of Chemistry 2024

Supporting Information

A Constitutional Isomer Selective Chemical Proteomic Strategy for System-wide Profiling of Protein Lysine 5-Hydroxylation

Yi-Cheng Sin[ab], Meeyeon Park[a], Timothy J. Griffin[a], Jeongsik Yong*[a], Yue Chen*[a]

a. Department of Biochemistry, Molecular Biology and Biophysics, the University of Minnesota at Twin Cities, Minneapolis, Minnesota, USA

b. Bioinformatics and Computational Biology Program, the University of Minnesota at Twin Cities, Minneapolis, Minnesota, USA

*Correspondence: Dr. Yue Chen (yuechen@umn.edu) or Dr. Jeongsik Yong (jyong@umn.edu)

Supporting Information

Supplementary Figures S1-S10

Figure S1. Mass spectra for the analysis of 1-aminopentan-2-ol, 1-aminopentan-3-ol, and 5-amino-2-pentanol.

Figure S2. Schematic workflow and mass spectra for the analysis of collagen 5-Hyl peptide.

Figure S3. MS/MS neutral loss fragmentation pattern of 5-Hyl peptide ion.

Figure S4. MS/MS spectrum comparison of D_3- or H_3- methoxyamine conjugated peptides with in vivo HMGN1 5-hydroxylysine modified peptide as example.

Figure S5. Schematic workflow and replicate analysis of 5-Hyl proteins with enzyme overexpression.

Figure S6. Venn diagram comparison of hydroxylysine identification between this study and previous study.

Figure S7. Example mass spectra for the identification of histone 5-Hyl peptides.

Figure S8. Statistical analysis of label-free quantification of 5-Hyl peptides.

Figure S9. Motif enrichment analysis of 5-Hyl sites identified from the nuclear lysates of control cells or cells overexpressing either short or long form of JMJD6.

Figure S10. Schematic workflow of the chemical pulldown assay for Western blotting and input Western blotting for selected proteins.

Figure S11. Mass spectra for the identification of 5-Hyl peptides on selected proteins.

Supplementary Tables S1-S5

Table S1. The list of 5-Hyl sites and proteins identified from the nuclear lysate proteins and normalized intensities with or without the expression of JMJD6 isoforms.

Table S2. The list of 5-Hyl sites and proteins identified from the acid extraction of 293T cells expressing short-form JMJD6.

Table S3. The list of O-GlcNAcylation sites and proteins identified in the raw data of 5-Hyl protein analysis from the nuclear lysates of 293T cells.

Table S4. Significantly enriched pathways and Gene Ontology among lysine 5-hydroxylation proteome in 293T nuclear lysate analysis.

Table S5. Significantly up- or down-regulated 5-Hyl sites comparing the overexpression of JMJD6 short-form and long-form in 293T cells.

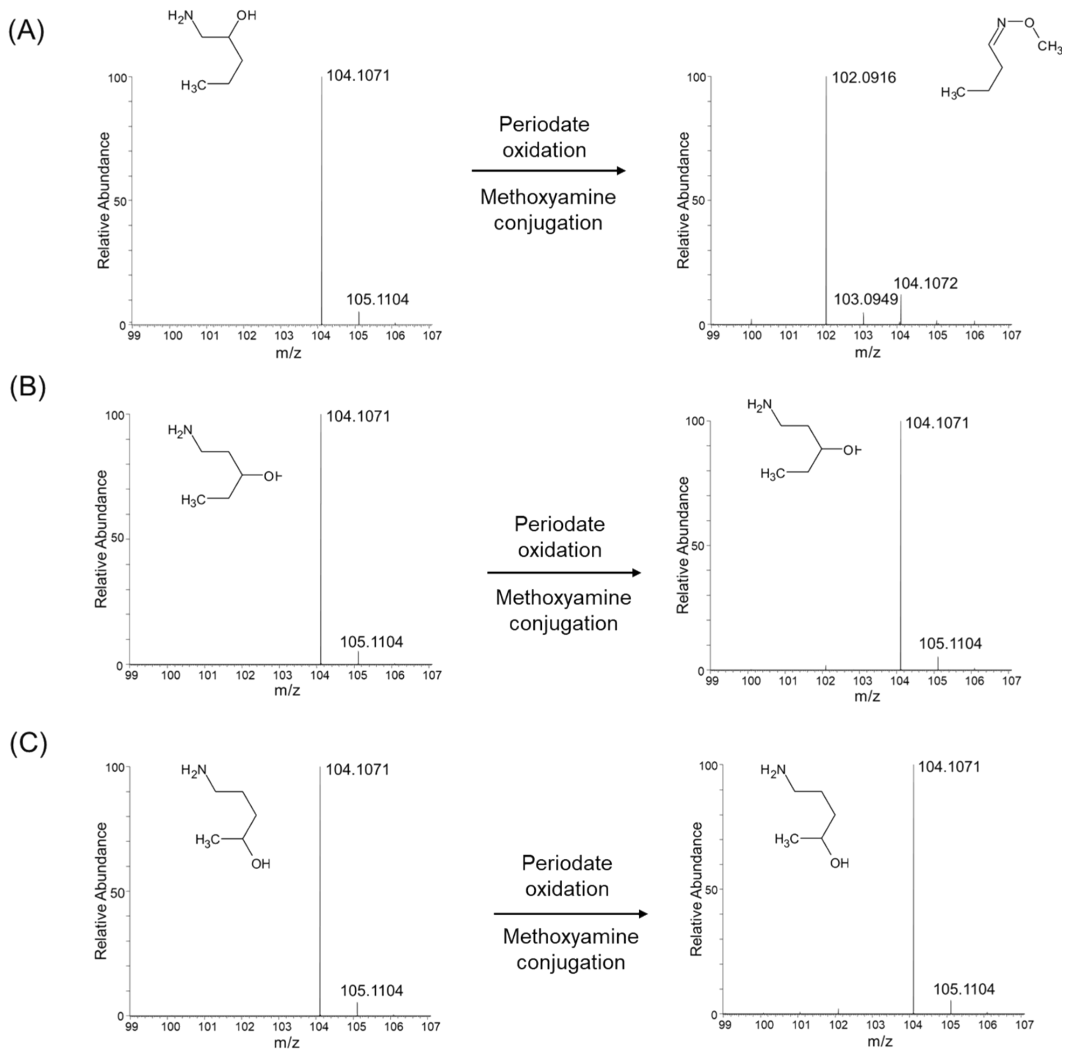

Figure S1. Mass spectra for the analysis of 1-aminopentan-2-ol, 1-aminopentan-3-ol, and 5-amino-2-pentanol. High resolution MS1 spectra of (A) 1-aminopentan-2-ol (B) 1-aminopentan-3-ol and (C) 5-amino-2-pentanol before and after the chemical reactions with periodate and methoxyamine.

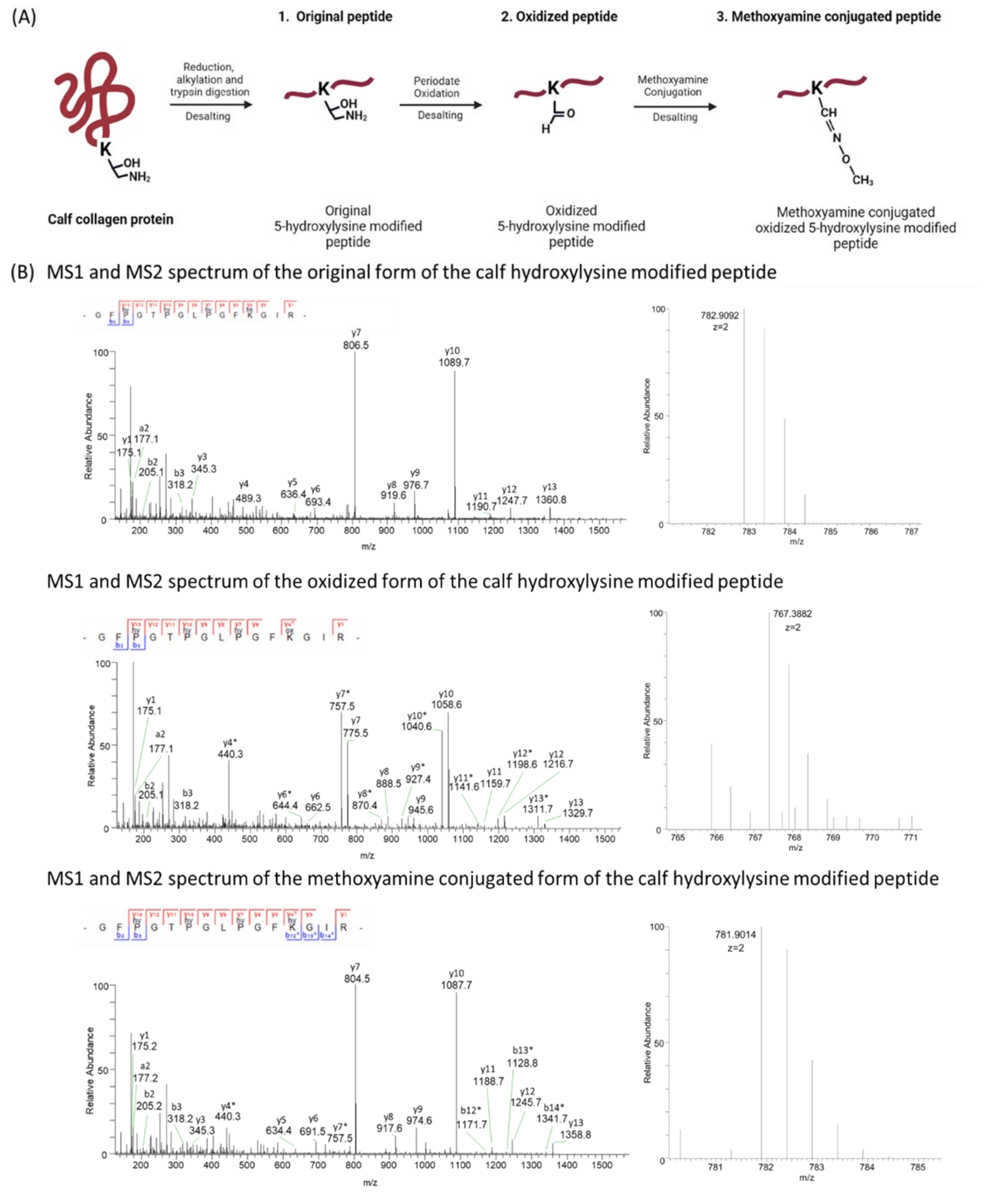

Figure S2 Schematic workflow and mass spectra for the analysis of collagen 5-Hyl peptide (A) Experiment workflow to evaluate the reaction efficiency through digested calf collagen protein. (B) MS2 fragmentation (left) and MS1 precursor (right) spectra of the collagen tryptic peptide "GFPGTPGLPGFKGIR" with no reaction (top panel), periodate oxidation reaction (middle panel) and methoxyamine conjugation reaction (lower panel). 'hy' on proline indicated hydroxyproline modification with mass shift of "O". 'hk' on lysine indicated original hydroxylysine modification with mass shift of "O". 'ox' on lysine indicated oxidized hydroxylysine modification with mass shift of "C(-1)H(-5)N(-1)O" and neutral loss of "H(2)O". 'hy' on lysine indicated methoxyamine conjugated oxidized hydroxylysine modification with mass shift of "H(-2)O" and neutral loss of "CH(5)NO". '*' indicated a neutral loss signal.

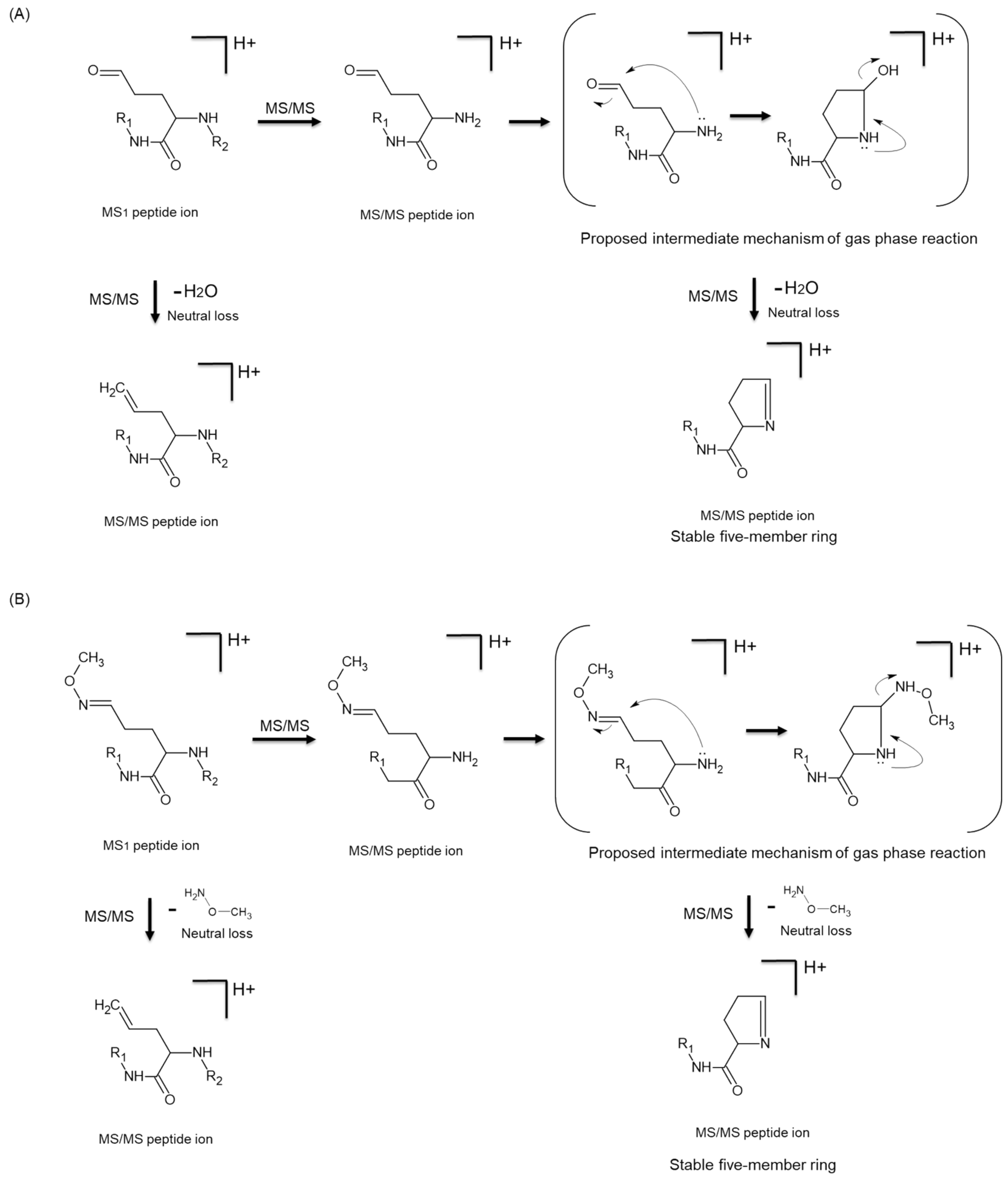

Figure S3. Proposed MS/MS neutral loss fragmentation mechanism of 5-Hyl peptide ion. (A) Oxidized 5-Hyl containing peptide. (B) Oxidized 5-Hyl containing peptide with methoxyamine conjugation.

(A)

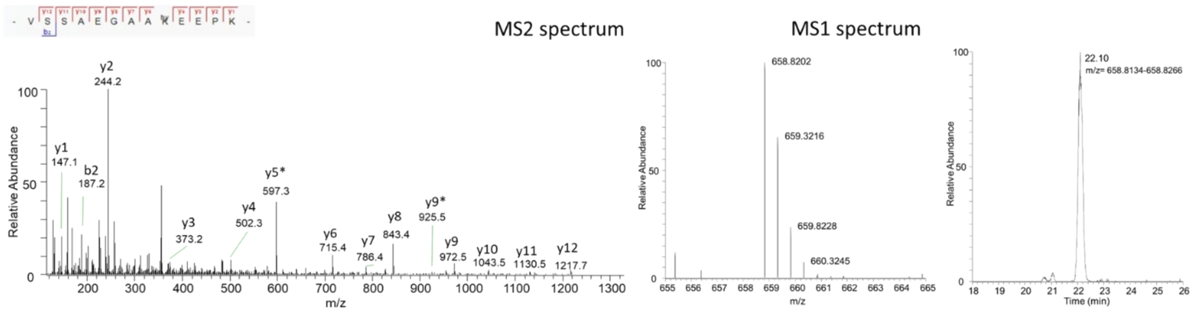

(B)

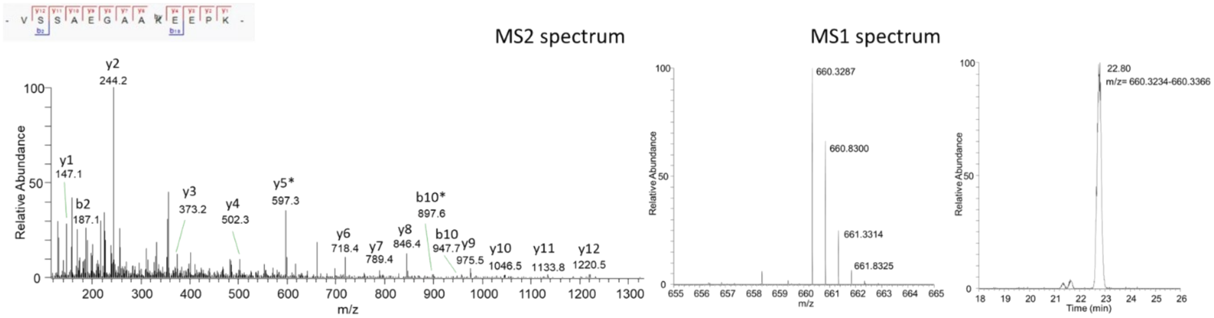

Figure S4. MS/MS spectrum comparison of D_3- or H_3- methoxyamine conjugated peptides with in vivo HMGN1 5-hydroxylysine modified peptide as example. (A) Methoxyamine conjugated peptide. "*" indicated the neutral loss signal of CH(5)NO. (B) D_3-Methoxyamine conjugated peptide. "*" indicated the neutral loss signal of CH(2)D(3)NO.

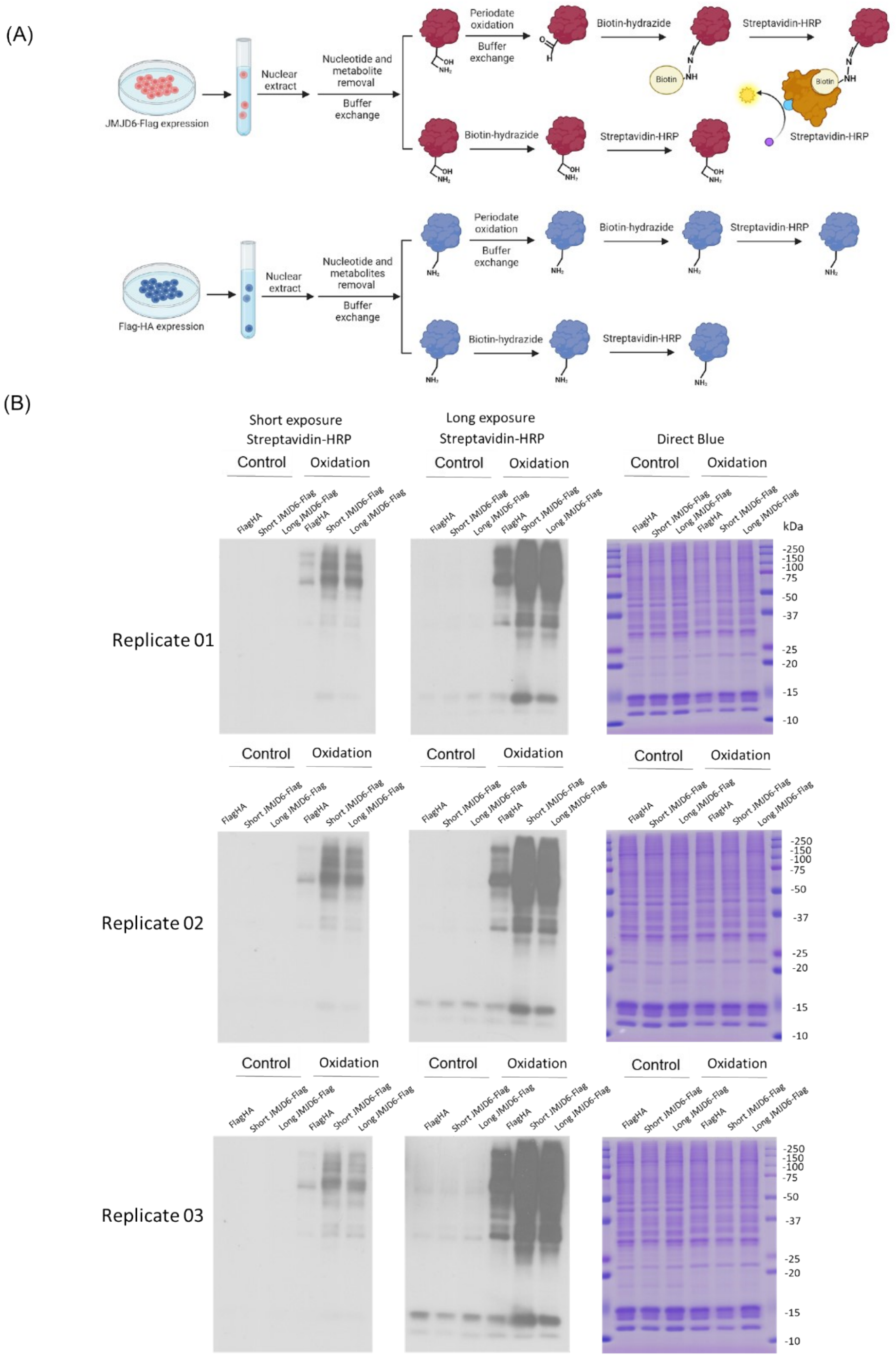

Figure S5. Schematic workflow and replicate analysis of 5-Hyl proteins with enzyme overexpression. (A) Experiment workflow to validate 5-Hyl protein abundance changes through biotin hydrazide and streptavidin blotting. (B) Biological triplicate analysis of streptavidin blotting on nuclear lysates with or without periodate oxidation followed by biotin hydrazide conjugation.

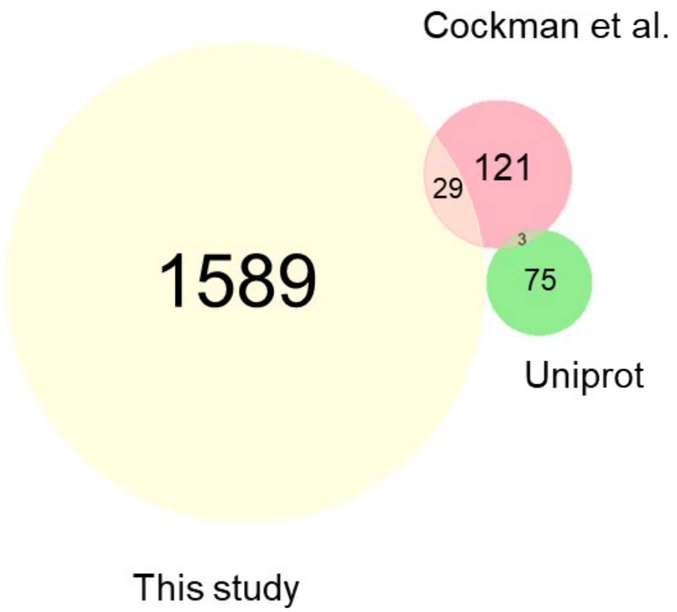

Figure S6. Venn diagram comparison of hydroxylysine between this study and previous study. Data included the identified 5-hydroxylysine modification sites in this study and annotated in the Uniprot database (http://www.uniprot.org) and hydroxylysine modification site identified in Cockman et al.

(A) H1F0_hydroxylated spectrum

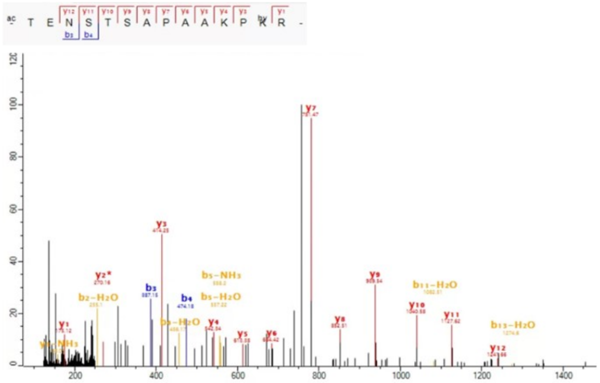

(B) HISTH1E_hydroxylated spectrum

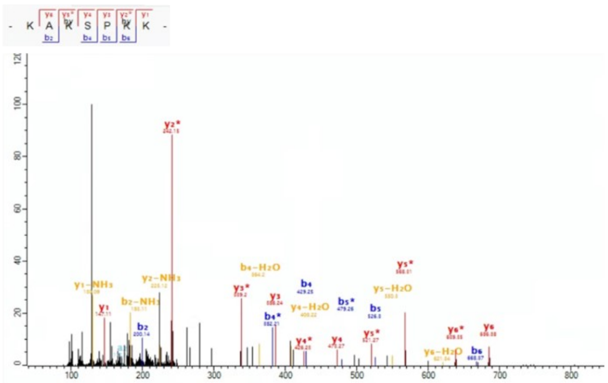

(C) HISTH1E_hydroxylated spectrum

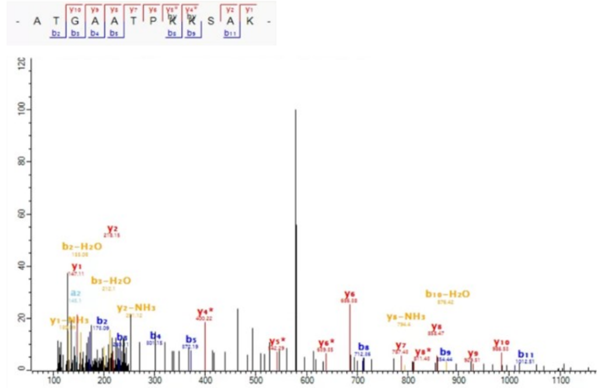

(D) H2AFX_hydroxylated spectrum

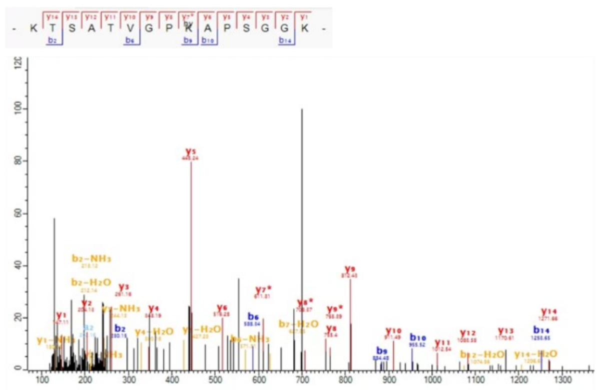

(E) HIST1H2BK_hydroxylated spectrum

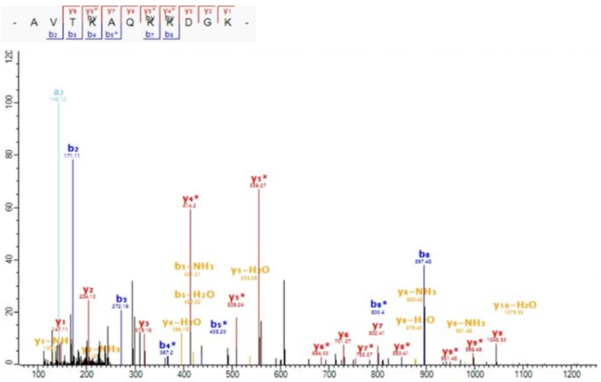

(F) HIST1H2BK_hydroxylated spectrum

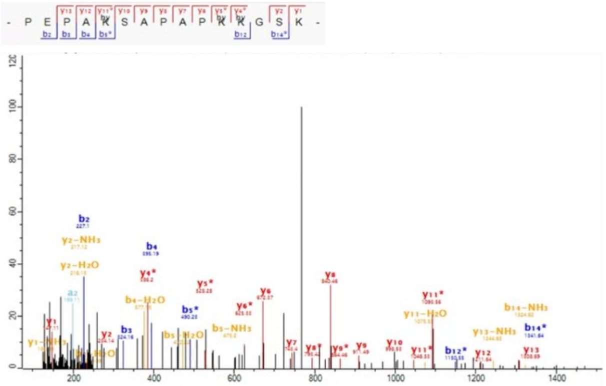

(G) HIST1H2BK_hydroxylated spectrum

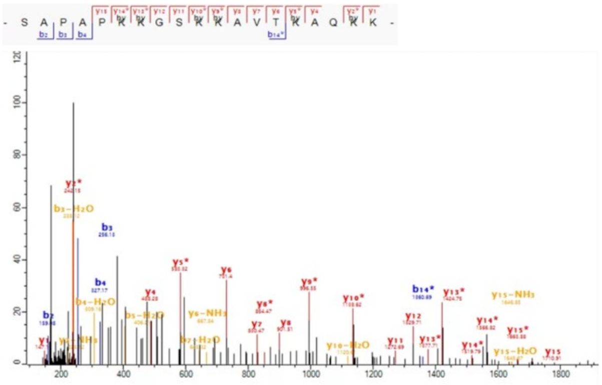

(H) HIST1H2BK_hydroxylated spectrum

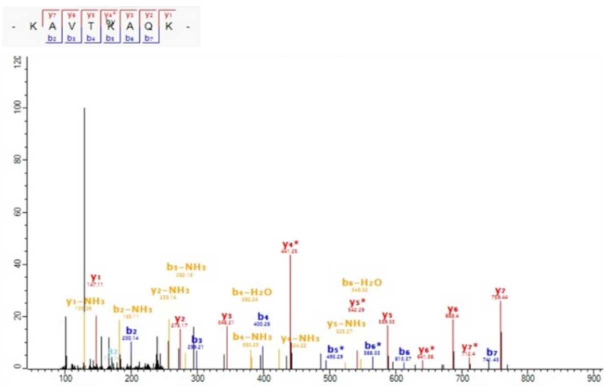

(I) HIST1H2BK_hydroxylated spectrum

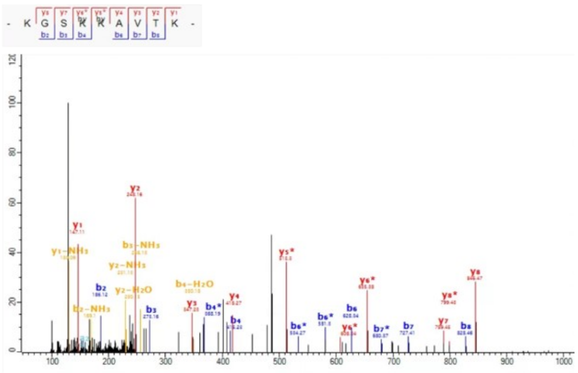

(J) HIST1H2BK_hydroxylated spectrum

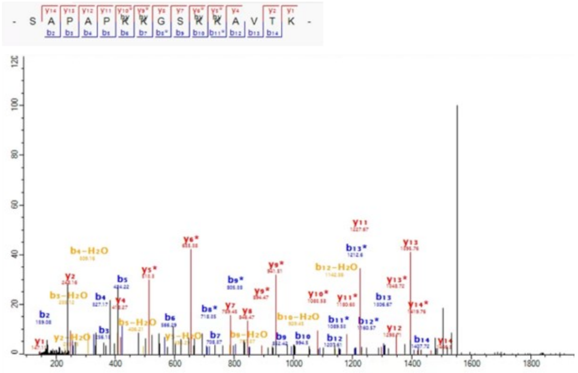

(K) HIST1H2BK_hydroxylated spectrum

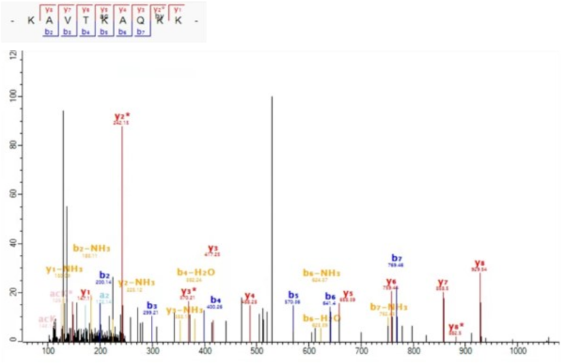

Figure S7. Example mass spectra for the identification of histone 5-Hyl peptides. ‘Hy’ indicates methoxyamine-conjugated oxidized 5-Hyl modification. ‘*’ indicates the neutral loss of methoxyamine conjugated oxidized 5-Hyl modification. ‘ac’ indicates acetylation.

(A)

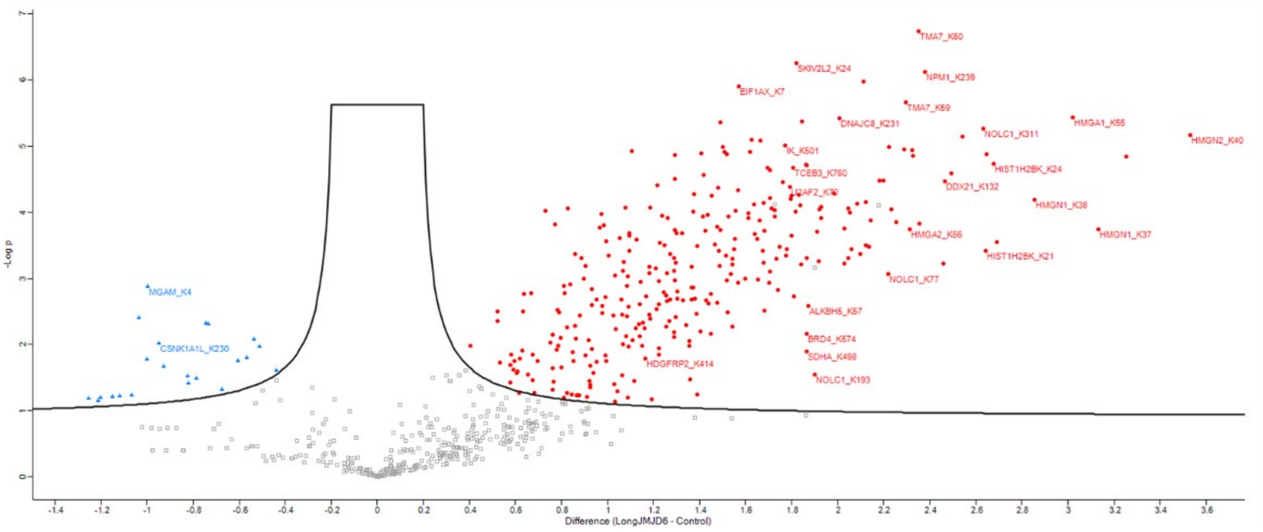

(B)

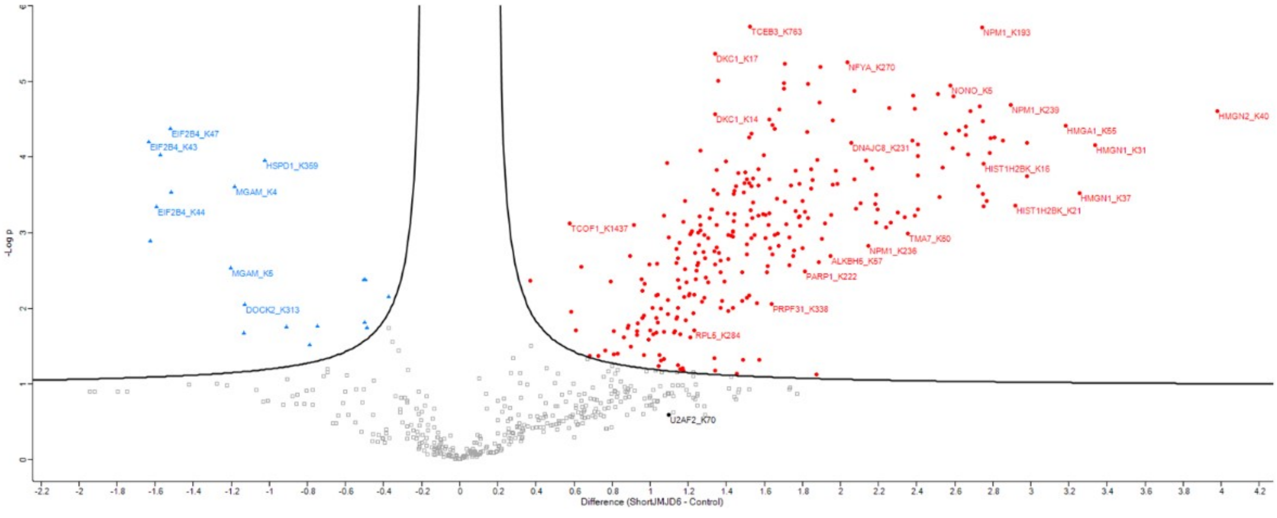

Figure S8. Statistical analysis of label-free quantification of 5-Hyl peptides. Volcano plots and statistical analysis of the label-free quantification of 5-Hyl sites mediated by the expression of (A) long form flag-JMJD6 expression or (B) short form flag-JMJD6 overexpression. Statistical analysis was performed with two-samples Student's T test with the permutation-based FDR cutoff of 0.05.

Control

motif	score	fg.matches	fg.size	bg.matches	bg.size	fold.increase
...............KK.K............	319.047518	41	394	6309	634755	10.469676
.........K.....K.K.............	316.871137	30	353	5015	628446	10.649852
..............KK...............	15.65356	73	323	49004	623431	2.875261
...............K...K...........	12.013029	55	250	43516	574427	2.90408
............M..K...............	9.315648	22	195	11569	530911	5.177427
...............K..K............	8.402083	36	173	36265	519342	2.98004
............K..K...............	8.9863	34	137	37426	483077	3.203327
...............K.........K.....	6.121407	23	103	30966	445651	3.213663

5-Hydroxylysine site motifs with the expression of short-form JMJD6

motif	score	fg.matches	fg.size	bg.matches	bg.size	fold.increase
...............KK.K............	615.305311	101	861	6309	634755	11.802223
............K.KK.K.............	327.488855	29	760	1067	628446	22.474392
...............K...KK..........	319.781235	54	731	5085	627379	9.114134
..............KK......K........	316.828041	41	677	4466	622294	8.438633
...............K..K..K.........	321.616019	47	636	4210	617828	10.844917
...............KK......K.......	315.275982	35	589	3870	613618	9.42193
............K..KK..............	314.882156	27	554	3722	609748	7.984138
..............KK...............	307.652656	97	527	42366	606026	2.632903
..............PK.K.............	313.968169	20	430	2350	563660	11.156061
...............K..K............	13.925206	73	410	36718	561310	2.721841
...............KK..............	14.238586	63	337	32538	524592	3.013988
............K..K...............	10.493872	55	274	37212	492054	2.65425
...............K......K........	7.398048	40	219	31941	454842	2.600926

5-Hydroxylysine site motifs with the expression of long-form JMJD6

motif	score	fg.matches	fg.size	bg.matches	bg.size	fold.increase
...............KK.K............	615.305311	93	845	6309	634755	11.073167
..............KK...K...........	319.631171	55	752	5065	628446	9.074723
............K..K....K..........	315.887674	41	697	4679	623381	7.837032
...............K.K....K........	317.583192	41	656	4736	618702	8.164881
...............KK.......K......	314.817854	32	615	3539	613966	9.0269
...............K..K..K.........	318.812646	39	583	4139	610427	9.865847
............K..K.K.............	314.426271	28	544	4108	606288	7.596397
...............KK..............	307.652656	95	516	39020	602180	2.841273
...............K..K............	15.65356	81	421	39543	563160	2.740092
..............KK...............	13.790237	66	340	35755	523617	2.842772
...............K...K...........	10.129158	53	274	35264	487862	2.676028
...............K..R............	7.098289	36	221	27397	452598	2.691038
............K..K...............	6.499008	32	185	27254	425201	2.698623

Figure S9. Motif enrichment analysis of 5-Hyl sites identified from the nuclear lysates of control cells or cells overexpressing either short or long form of JMJD6.

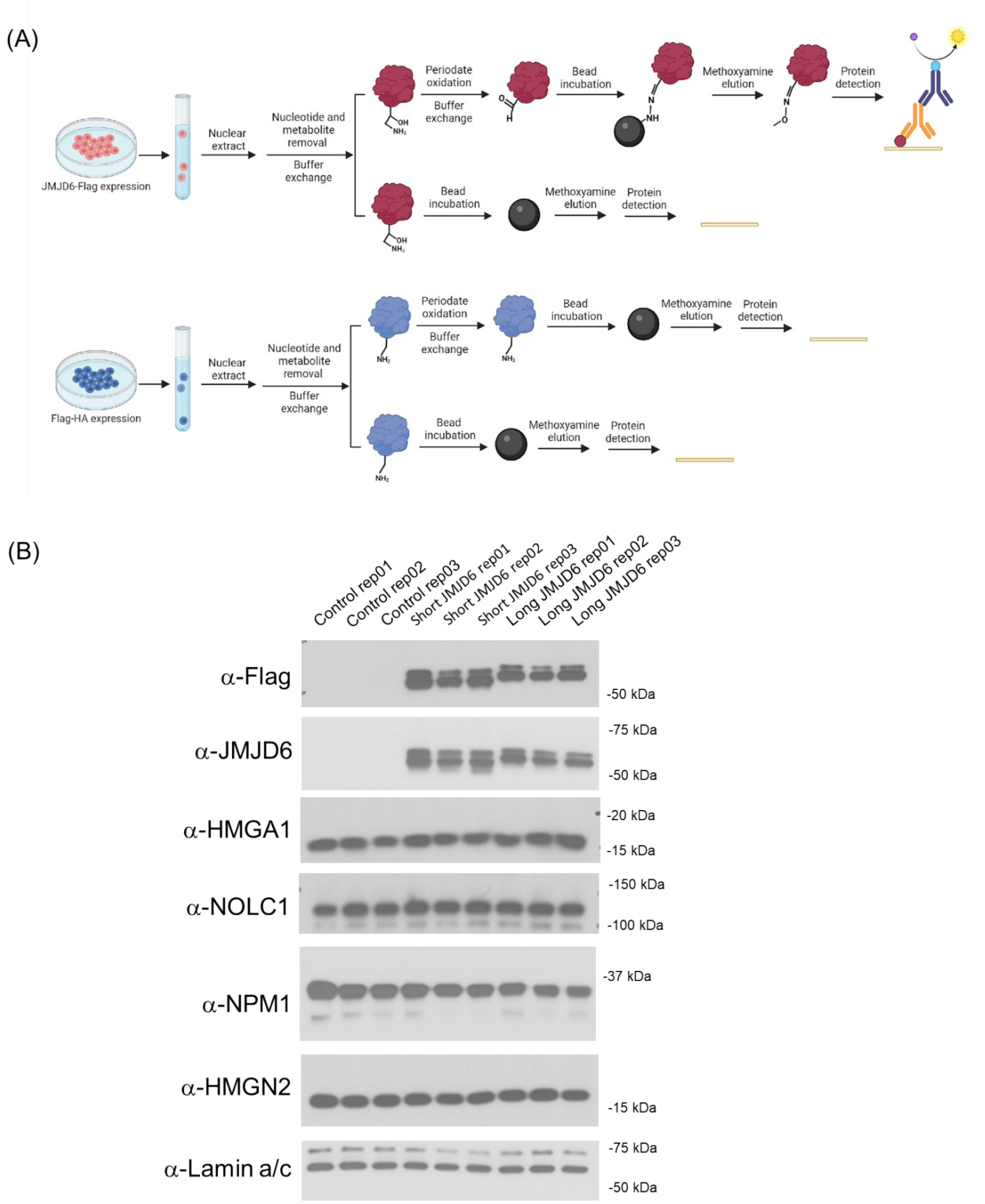

Figure S10. Schematic workflow of the chemical pulldown assay for Western blotting and input Western blotting for selected proteins. (A) Experiment workflow for chemical pulldown assay and (B) Western blotting of the inputs of all the experiment groups.

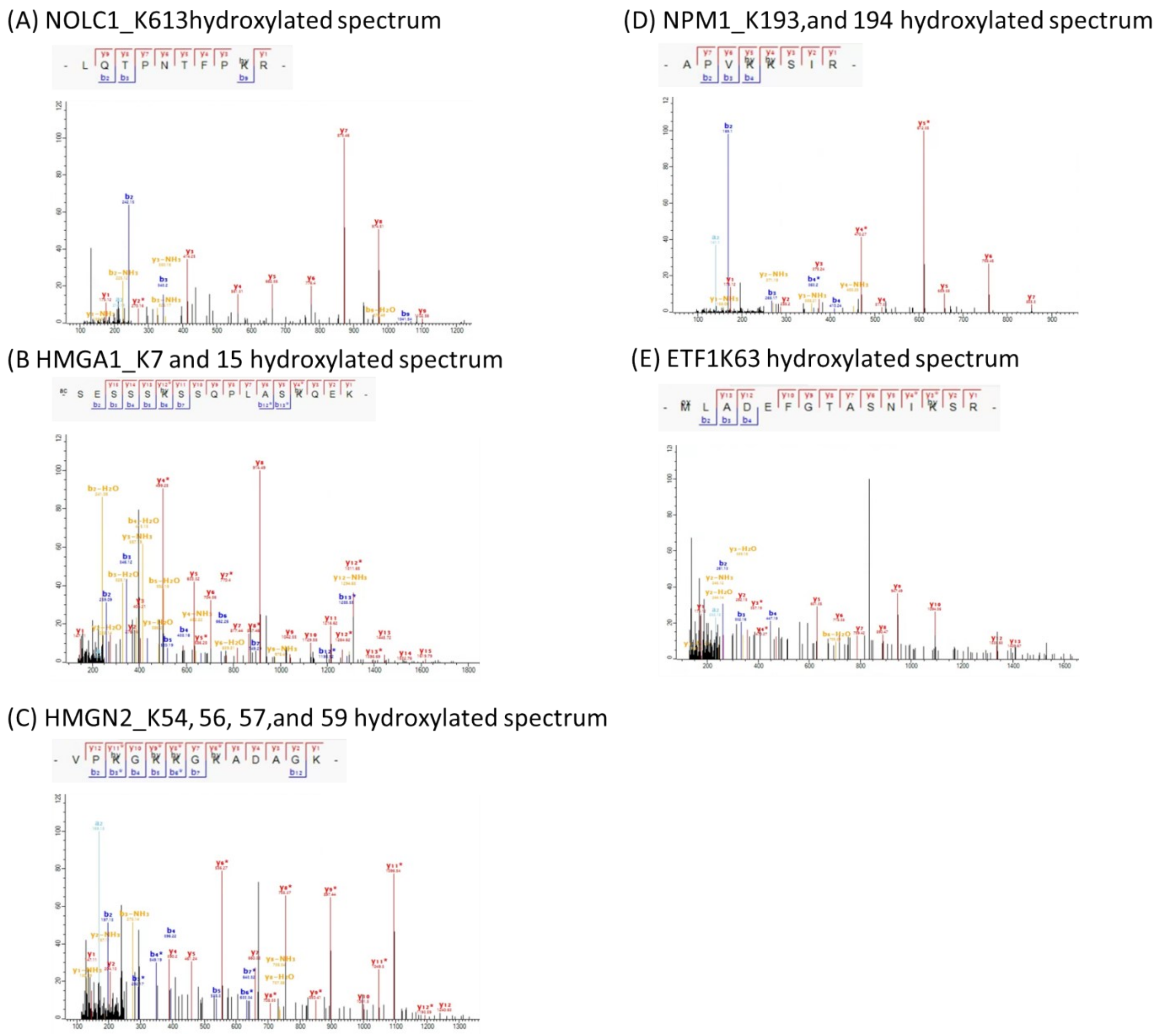

Figure S11. Mass spectra for the identification of 5-Hyl peptides on selected proteins. ‘Hy’ indicates methoxyamine-conjugated oxidized 5-Hyl modification. ‘*’ indicates the neutral loss of methoxyamine conjugated oxidized 5-Hyl modification.
